# Automated Quality Assurance of Online NIR Analysers

**DOI:** 10.1155/JAMMC.2005.44

**Published:** 2005

**Authors:** Kari Aaljoki

**Affiliations:** 1 Neste Jacobs Oy P.O. Box 310 Porvoo 06101 Finland

## Abstract

Modern NIR analysers produce valuable data for closed-loop process control and optimisation practically in real time. Thus it is highly important to keep them in the best possible shape. Quality assurance (QA) of NIR analysers is an interesting and complex issue because it is not only the instrument and sample handling that has to be monitored. At the same time, validity of prediction models has to be assured. A system for fully automated QA of NIR analysers is described. The system takes care of collecting and organising spectra from various instruments, relevant laboratory, and process management system (PMS) data. Validation of spectra is based on simple diagnostics values derived from the spectra. Predictions are validated against laboratory (LIMS) or other online analyser results (collected from PMS). The system features automated alarming, reporting, trending, and charting functions for major key variables for easy visual inspection. Various textual and graphical reports are sent to maintenance people through email. The software was written with Borland Delphi 7 Enterprise. Oracle and PMS ODBC interfaces were used for accessing LIMS and PMS data using appropriate SQL queries. It will be shown that it is possible to take actions even before the quality of predictions is seriously affected, thus maximising the overall uptime of the instrument.


Journal of Automated Methods & Management in Chemistry, **2005** (2005), no. 2, 44–49
© 2005 Hindawi Publishing Corporation

### Automated Quality Assurance of Online NIR Analysers

Kari Aaljoki

*Neste Jacobs Oy, P.O. Box 310, 06101 Porvoo, Finland*
*Email: kari.aaljoki@fortum.com*

*Received 31 August 2004; Accepted 17 September 2004*

Modern NIR analysers produce valuable data for closed-loop process control and optimisation practically in real time. Thus it is highly important to keep them in the best possible shape. Quality assurance (QA) of NIR analysers is an interesting and complex issue because it is not only the instrument and sample handling that have to be monitored. At the same time, validity of prediction models has to be assured. A system for fully automated QA of NIR analysers is described. The system takes care of collecting and organising spectra from various instruments, relevant laboratory, and process management system (PMS) data. Validation of spectra is based on simple diagnostics values derived from the spectra. Predictions are validated against laboratory (LIMS) or other online analyser results (collected from PMS). The system features automated alarming, reporting, trending, and charting functions for major key variables for easy visual inspection. Various textual and graphical reports are sent to maintenance people through email. The software was written with Borland Delphi 7 Enterprise. Oracle and PMS ODBC interfaces were used for accessing LIMS and PMS data using appropriate SQL queries. It will be shown that it is possible to take actions even before the quality of predictions is seriously affected, thus maximising the overall uptime of the instrument.

### 1. INTRODUCTION

One of the most important issues of successful multivariate online analysis implementations is the strict validation of models and data used for predictions. Some spectral features can rather easily be used for checking basic sample handling and analyser performance. Similarly, for example, total area of the chromatogram, area of unidentified peaks, and peak retention times have been used for monitoring the performance of gas chromatographs [1].

Model validation is normally based on manually running some reference samples and comparing the results against laboratory results. This calls for good quality assurance (QA) programme and extra work. It obviously means also that the system will not be online during these operations. In contrast, this system collects reference data of routine schedule samples from LIMS (samples are taken to the laboratory from the same fast loop close to NIR cells, thus representing the same product) in order to minimise extra work. The other benefit of this approach is that it continuously follows product changes and tests the model in variable prediction space. For example, often at least two grades (“summer” and “winter”) of diesel and gasoline are produced to meet seasonal requirements.

QA of NIR application can be divided into four areas:

(i) sample handling;
(ii) analyser itself (repeatability and accuracy of spectra, S/N, and so on);
(iii) initial modelling (the coverage of calibration sample space, quality of input data, and so on);
(iv) long-term validation of predictions.

Problems in sample handling can lead to various scattering effects that will eventually have an impact on predictions and are very hard to eliminate precisely. On the other hand, evolving scattering effects are rather easy to see from spectra and can thus be detected with simple logic.

Analysers themselves can produce useful measures for diagnostics such as light intensity through sample and reference fibre.

Validation of predictions must naturally be based on laboratory measurements of the same sample. History of the prediction quality helps to see possible offsets and trends. Readily available historical data of other diagnostics helps to see if the measurement will be affected and corrective actions should be taken.

Although extensive manual procedures for the validation of multivariate quantitative analysis and process spectrophotometers have been described in detail [2, 3], they are not widely used because they are labour intensive. Therefore, one of the project goals was to implement an automated system thus saving valuable time of analyser maintenance personnel. The system, called OnQ for short, was designed for checking instrument and sample handling problems few times a day on the basis of information found from the spectra. It validates models immediately once the reference data becomes available in LIMS.

It should be mentioned that just recently, one related article has been published [4]. OnQ, however, goes much further in automation and requires no extra sampling thanks to its software link to LIMS. In addition, for example, email is used to distribute all automated alarms, notes, reports, trends, and SPC charts.

### *Database*

OnQ depends heavily on dynamic data. Thus a natural choice is to use a database for storing system and collected data. Database defines the analysers, several cross reference tables, and the data collected from the analysers, laboratory LIMS, and process computer. Some of its information is also used in building LIMS and PMS SQL queries. Some tables are used for storing information about various events such as alarm notifications to help system logic to avoid sending messages of recurring situations. For example, OnQ does not notify the user if consecutive spectra indicate a sample handling problem. It gives the maintenance personnel a defined time for fixing the problem before notifying again about the same problem. A message will be sent, however, if the problem becomes worse (different problems are associated to different severity levels).

Currently, the database consists of 28 tables. Only the most important ones are briefly described here.

### *Major tables*

(a) *Analyser:*
  (i) analyser ID and channel number translates to process unit and stream for SQL query;
  (ii) type defines how its data file is parsed (various instruments produce different file formats);
  (iii) currently 23 records.

(b) *User:* defines the user, his/her email address, and cellular phone number.

(c) *Maintenance:* defines who (user) is responsible for a given analyser and thus to whom email will be sent in case of event passing a given severity level.

(d) *Recipe:* defines min, max, max move (difference between consecutive measurements), and max difference for each measurement analyser/channel by analyser/channel.

(e) *OnQSpectra:* collected spectral data such as baseline, absorbance maximum, sample light, and reference light (currently, data from close to 500 000 spectra have been collected).

(f) *ChannelStatus:* tells the current status of the analyser channel by channel.

(g) *RefResults:* lab reference results, corresponding predictions, and online results, if any, at the same sample time (the table holds currently over 28 000 entries).

(h) *Backlog:* lists of all the outstanding (lab testing unfinished) laboratory samples.

(i) *SpectrumSpec:* defines the criteria for spectrum validity evaluation analyser/channel by analyser/channel.

(j) *Events:* lists all time stamped deviations from normal operation.

(k) *MailLogi:* keeps time stamped track of mail sent by analyser/channel (helps to avoid sending repeated messages).

(l) *Alarms:* pending events requiring user verification.

### *Software modules*

Technically speaking, OnQ has been built using Borland Delphi 5 (upgraded later with minor modifications to Delphi 7) and it runs on Windows NT/2000/XP. OnQ uses FlashFiler (TurboPower, now open source project at www.sourceforge.net) as its database engine. Laboratory data from in-house-built Oracle 7.3-based LIMS running on HP9000 is collected using SQL queries. Some reference values from other online analysers are collected from ABB's process management system (PMS) using its ODBC capabilities. SPC charts and trends were written using TeeChart (www.steema.com) components. These visual reports are distributed as JPG or as smaller-size native TeeChart files. The advantage of the last format is that in this case, the files are much smaller and the user has a full control of the graphical reports with a free TeeChartOffice (www.steema.com) programme. MS Excel and Word were used as COM servers for producing various reports that are mailed as spreadsheets or as documents, e.g., to maintenance people. Other Delphi add-on components that were used extensively came originally from TurboPower: Orpheus, SysTools, OfficePartner, and ShellShock (now www.sourceforge.net).

The major modules with a brief description about their purpose are listed in logical or execution order. The logic of the three first ones is depicted in Figures 1 and 2.

(a) *Filecollector:*
  (i) collects the spectra from various NIR analysers to OnQ new files directory;
  (ii) in some cases, deletes unnecessary extra files from analyser PC, thus helping its housekeeping.

(b) *SpectralMonitor:*
  (i) checks the quality of spectra. Mails diagnostics report to the maintenance if a problem is found. An expert's advice how to fix the problem is included as an email attachment;
  (ii) checks also if a reference sample has been taken to the lab (builds and executes an appropriate SQL query). If a reference sample has been taken, the spectrum is renamed and stored to an analyser/channel specific folder for model validation and possible later model updates just to save the modeller's time; otherwise, the spectrum is discarded after storing its diagnostic variables in the database.

(c) *BacklogMonitor:*
  (i) monitors missing laboratory results; validates predictions against laboratory results;
  (ii) mails diagnostics report to the maintenance if exceeding difference is found.

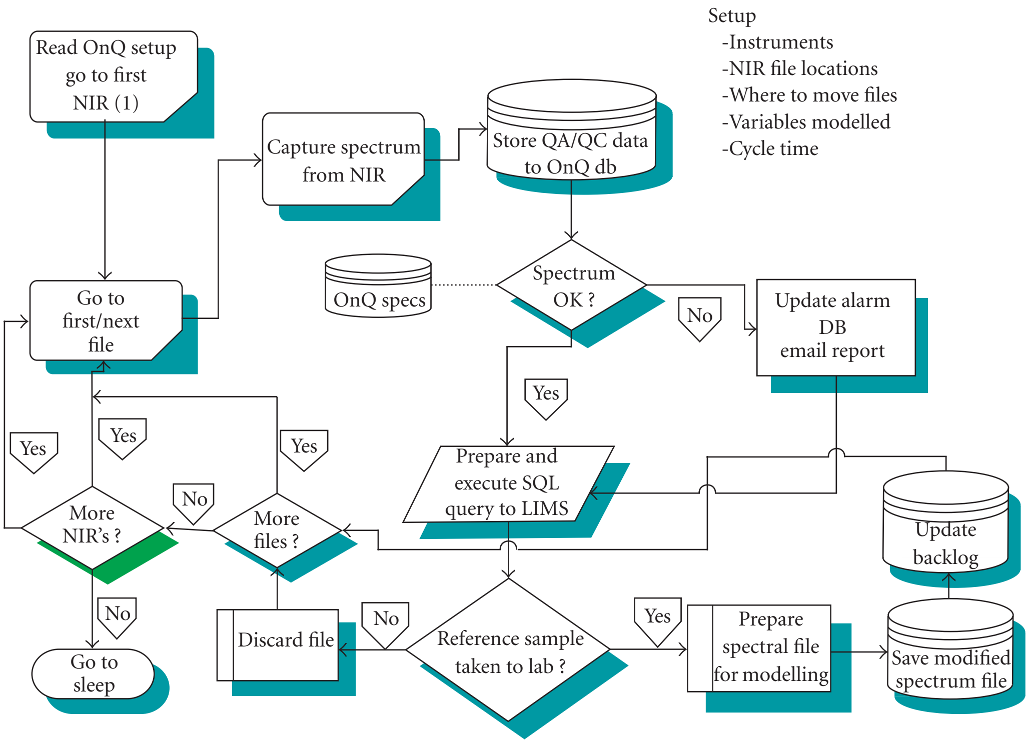

FIGURE 1: Flowchart of NewFilesMonitor + SpectralMonitor.

(d) *TrendsAndCharts:*
  (i) programme for creating trends and SPC charts of selected diagnostics/predictions. Graphics results can be saved and mailed to customers. The use of native TeeChart format has some advantages. The size of the graphics file is only about 10% of that in JPEG format and the user has a full control of the graphical report with the free TeeChartOffice programme;
  (ii) command line is parsed for options (or macro file) if executed by another program.

(e) *Reporter:*
  (i) programme for creating reports of selected diagnostics/predictions; uses MS Excel and Word as COM servers for creating report files that are mailed to maintenance personnel;
  (ii) command line is parsed for options (or macro file) if executed by another program.

(f) *Scheduler:* used for scheduling trends, SPC charts, and reports with predefined set of variable definitions (simple macro file). Usually the most important diagnostics variables are selected for monitoring; others are available using the previously mentioned modules.

The modules have written to support both interactive use and execution by another program. Normally Scheduler, which will pass special instructions to them for doing given tasks, is used for scheduling them from usually a few times a day. Some, for example, trending tasks, which are used for looking for long-term changes, are scheduled only a few times a week, because we want to avoid putting too much burden on the users by sending too much information. Model validation trends and charts are scheduled after laboratory sample timetables.

### 2. EXAMPLES

This paper shows some simple examples taken from real situations in order to demonstrate the usefulness of automated QA and analyser validation.

### 2.1. *QA of spectra*

A trend of spectrometer sample light and reference light intensity, examples of simple diagnostics variables, is shown in Figure 3.

The rules to take actions in this case are as follows.

(i) Send an alarm message to the user if the reference light intensity drops too rapidly or below the specified level (defined for each analyzer and channel). The reason for this failure is most probably a lamp reaching its lifetime (we have seen only one case of broken reference fibre). One case can be seen in Figure 3.

(ii) Send an alarm message to the user if the sample light intensity drops too rapidly or below the specified level (defined for each analyzer and channel). The first analyser item to be checked in this case is sample handling

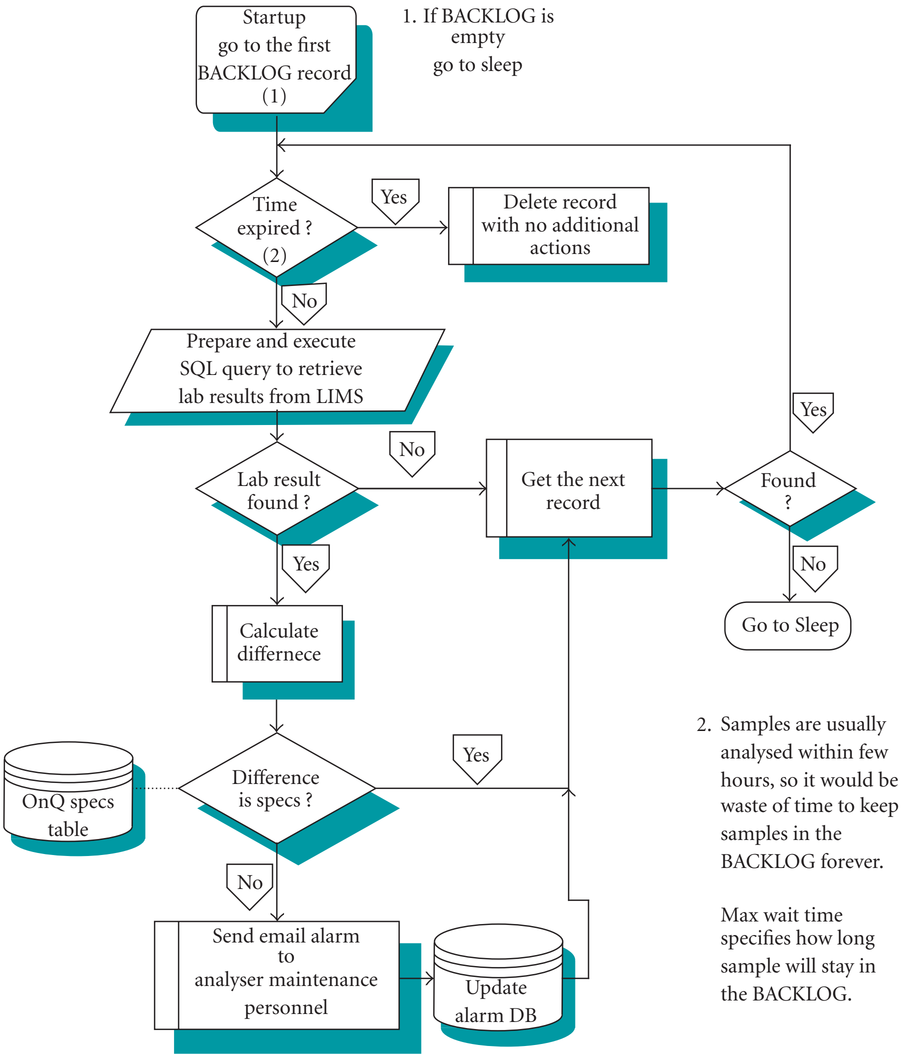

Figure 2: Flowchart of BacklogMonitor.

if the reference light intensity rule was passed (both will go down if the lamp fails). Again, one case can be seen in Figure 3.

The second example illustrates a situation where baseline level has started to fluctuate. This is normally an indication of dirty sample cell, and sample cell and, for example, sample filters call for cleaning. Occasional peaks in Figure 4 are related to very small catalyst particles passing through failing sampling system.

It is also possible to monitor some variables that are related to the instrument hardware itself such as the temperature inside the analyser cabinet (Figure 5).

### 2.2. *Validation of models*

Trending both predictions and reference results (Figure 6) is an easy visual way to see if the model works well. Viewing trends like this helps to identify systematic changes and to see when it is time to update the model. As mentioned, BacklogMonitor sends an alarm when the prediction difference exceeds its maximum limit. In case of such an alarm, the user is advised to view corresponding trend or SPC chart.

SPC charts provide a more statistically based tool for model validation. OnQ uses individuals control charts [5] of prediction differences (lab-prediction) for this purpose (Figure 7). Tests for out-of-control situations are currently, however, left to the user.

### 3. CONCLUSIONS

Automated QA system helps maintenance people to act sometimes even before predictions start to fail (e.g., spectrum becomes an outlier). It has resulted in shorter response times in maintenance actions. Thus the average analyser uptime has increased, thus helping in maximising the benefits due to closed-loop control and process optimisation.

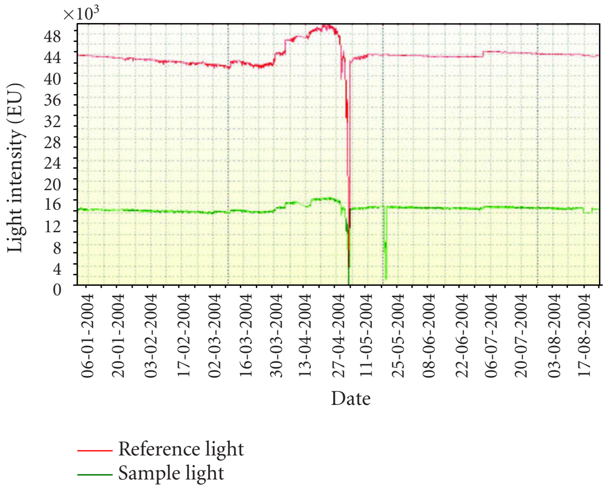

FIGURE 3: OnQ diagnostic variable trend(s) of sample light and reference light intensity (default data range is from the beginning of the current year until current date). NIR: ID 5020 channel 6 process: unit VK stream CDIZ.

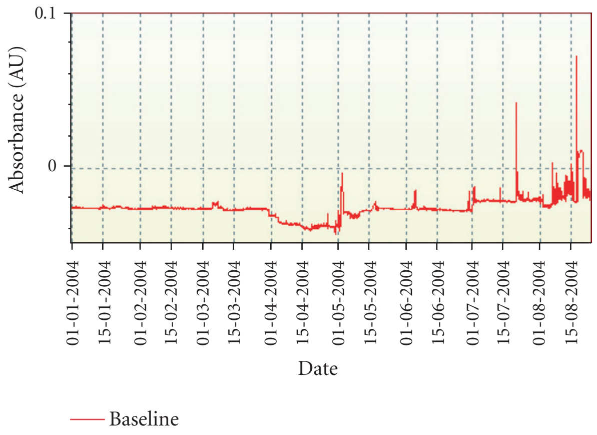

FIGURE 4: OnQ diagnostic variable trend(s) of baseline (minimum absorbance). NIR: ID 5020 channel 3 process: unit VKT stream STRVKT.

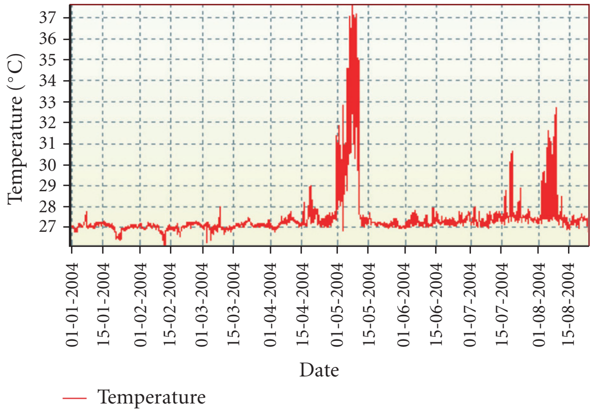

FIGURE 5: OnQ diagnostic variable trend(s) of an analyser cabinet temperature. NIR: ID 5020 channel 3 process: unit VKT stream STRVKT.

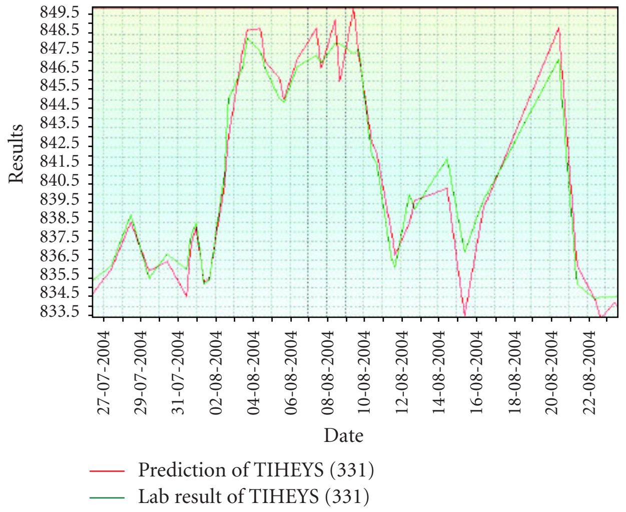

FIGURE 6: OnQ trend(s) of predictions/lab results. Monitoring model performance with a trend. NIR: ID 5020 channel 3 process: unit VKT stream STRVKT.

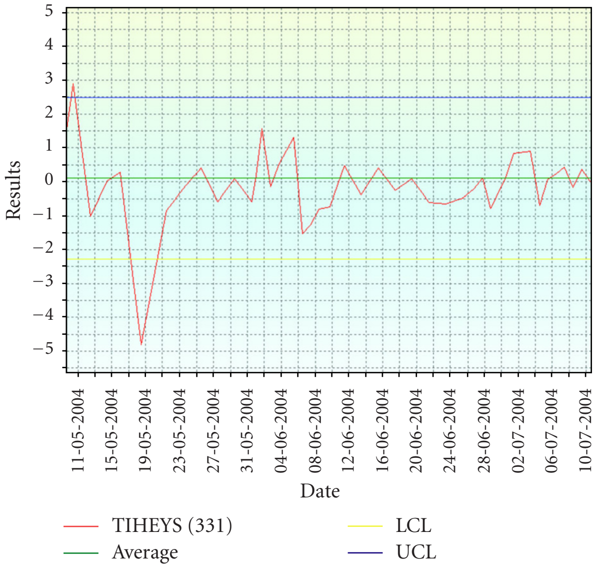

FIGURE 7: Example of individuals chart. OnQ SPC plot (individuals chart). NIR: ID 5020 channel 3 process: unit VKT stream STRVKT.

In addition to improving the overall quality of our online NIR analysers, automated QA has provided us with some extra valuable side benefits:

(i) automated capturing and archiving of reference spectra for model updates;

(ii) improvements in the quality of laboratory reference measurements;

(iii) automatic disposal of garbage spectra or extra related files (improves MS Windows performance which may be affected when the number of spectra gets high ($>$ 100 000).

The success in phase I has motivated us to expand the system to incorporate also other types of online analysers in the near future.

### REFERENCES

[1] K. Aaljoki and N. Dressler, "An automated chromatography QA/QC system," *American Laboratory*, pp. 25–28, June 1997.

[2] ASTM, "Standard practice for validation of multivariate process infrared spectrophotometers," D 6122-97, 1997.

[3] ASTM, "Standard practices for infrared multivariate quantitative analysis," E 1655-99, 1999.

[4] J. M. Hays, R. L. Baird, and J. B. Cross, "Use automated quality assurance measurements for process analyzers," *Hydrocarbon Processing*, vol. 83, no. 6, pp. 75–78, 2004.

[5] W. H. McNeese and R. A. Klein, *Statistical Methods for the Process Industries*, ASQC Quality Press, Milwaukee, Wis, USA, 1991.

